# *N*-[(3,5-Dimethyl­pyrazol-1-yl)meth­yl]phthalimide

**DOI:** 10.1107/S160053680802518X

**Published:** 2008-08-09

**Authors:** Su-Qing Wang, Fang-Fang Jian, Huan-Qiang Liu

**Affiliations:** a Microscale Science Institute, Department of Chemistry and Chemical Engineering, Weifang University, Weifang 261061, People’s Republic of China; b Microscale Science Institute, Weifang University, Weifang 261061, People’s Republic of China; c Department of Chemistry and Chemical Engineering, Weifang University, Weifang 261061, People’s Republic of China

## Abstract

The title compound {systematic name: 2-[(3,5-dimenthylpyrazol-1-yl)meth­yl]isoindole-1,3-dione}, C_14_H_13_N_3_O_2_, was prepared by reaction of *N*-(bromo­meth­yl)phthalimide and 3,5-dimethyl­pyrazole in chloro­form solution. The mol­ecular structure and packing are stabilized by intra­molecular C—H⋯O hydrogen-bonding and C—H⋯π inter­actions.

## Related literature

For related literature, see: Jian *et al.* (2003 ▶, 2004 ▶); Barszcz *et al.* (2004 ▶).
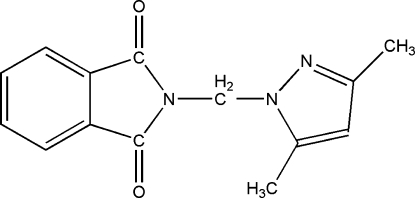


## Experimental

### 

#### Crystal data

C_14_H_13_N_3_O_2_
*M* *_r_* = 255.27
Monoclinic, 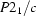

*a* = 12.285 (2) Å
*b* = 8.4576 (15) Å
*c* = 15.6162 (19) Å
β = 127.566 (8)°
*V* = 1286.1 (3) Å^3^
*Z* = 4
Mo *K*α radiation
μ = 0.09 mm^−1^
*T* = 293 (2) K
0.20 × 0.15 × 0.10 mm

#### Data collection

Bruker SMART CCD area-detector diffractometer
Absorption correction: none
8080 measured reflections
3090 independent reflections
1464 reflections with *I* > 2σ(*I*)
*R*_int_ = 0.060

#### Refinement

*R*[*F* ^2^ > 2σ(*F* ^2^)] = 0.056
*wR*(*F* ^2^) = 0.178
*S* = 0.98
3090 reflections
173 parameters
H-atom parameters constrained
Δρ_max_ = 0.33 e Å^−3^
Δρ_min_ = −0.21 e Å^−3^

### 

Data collection: *SMART* (Bruker, 1997 ▶); cell refinement: *SAINT* (Bruker, 1997 ▶); data reduction: *SAINT*; program(s) used to solve structure: *SHELXS97* (Sheldrick, 2008 ▶); program(s) used to refine structure: *SHELXL97* (Sheldrick, 2008 ▶); molecular graphics: *SHELXTL* (Sheldrick, 2008 ▶); software used to prepare material for publication: *SHELXTL*.

## Supplementary Material

Crystal structure: contains datablocks global, I. DOI: 10.1107/S160053680802518X/at2593sup1.cif

Structure factors: contains datablocks I. DOI: 10.1107/S160053680802518X/at2593Isup2.hkl

Additional supplementary materials:  crystallographic information; 3D view; checkCIF report

## Figures and Tables

**Table 1:** Hydrogen-bond geometry (Å, °)

*D*—H⋯*A*	*D*—H	H⋯*A*	*D*⋯*A*	*D*—H⋯*A*
C6—H6*B*⋯O1	0.97	2.58	2.917 (3)	101
C11—H11*A*⋯*Cg*2^i^	0.93	2.96	3.723 (3)	140

Symmetry code: (i) 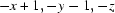. *Cg*2 is the centroid of atoms N2,N3,C2–C4.

**Fig. 1.**
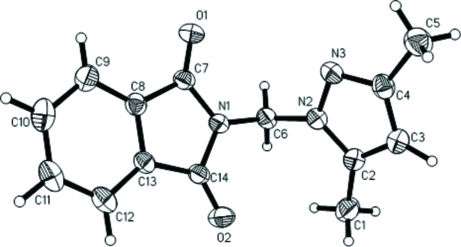
The molecular structure and atom-labeling scheme for (I), with displacement ellipsoids drawn at the 30% probability level.

## supplementary crystallographic information

### Comment

The 3,5-dimethyl pyrazole and its derivatives are of considerable interest as the ligands in many biological systems in which they procvide the potential binding site for metal ions (Barszcz *et al.*, 2004). In our search for new ligands of this type, we have synthesized the title compound  (I), and describe its structure here.

In the crystal structure of (I) (Fig. 1), the C═O bond length [1.206 (3) Å), (1.208 (3) Å] and the C—N bond length [1.397 (2) Å), (1.396 (3) Å] (Table 1) are in agreement with those observed before (Jian *et al.*, 2004; Jian *et al.*, 2003). The dihedral angle formed by the ring A (N1/C7/C8/C13/C14) and the ring C (C8–C13) is 1.3 (0)°. The dihedral angles formed by the ring A and ring C with the ring B (N2/N3/C2–C4) are 72.0 (1) and 72.0 (4)°, respectively. There is a C—H···O intramolecular interaction (see table 2). The molecular structure is also stabilized by intermolecular C—H···π interactions (Table 2).

### Experimental

*N*-bromomethyl phthalic imidine 7.2 g (0.03 mol) and 3,5-dimethyl pyrazole 2.88 g (0.03 mol) were dissolved in 30 ml chloroform. The solution was cooled to 283 K. Then, 4.4 ml (0.03 mol) triethylamine was added dropwise *via* cannula into the well stirred solution The reaction mixture was stirred at 283 K for 6 h. Then the solution was continued to stir at room temperature about 17 h. 20 ml water was added into the solution, the organic phase was seperated and dryed with anhydrous potassium carbonate, The colourless organic phase was evaporated. The title compound is afforded in 65% yield. The colourless crystals of suitable for X-ray determination were obtained from anhydrous ethanol at room temperature after two days.

### Refinement

H atoms were fixed geometrically and allowed to ride on their parent atoms, with C—H = 0.93 - 0.97Å, and with *U*_iso_(H)=1.2 or 1.5*U*_eq_(C).

### Crystal data

**Table:**

C_14_H_13_N_3_O_2_	*F*_000_ = 536
*M**_r_* = 255.27	*D*_x_ = 1.318 Mg m^−^^3^
Monoclinic, *P*2_1_/*c*	Mo *K*α radiation λ = 0.71073 Å
Hall symbol: -P 2ybc	Cell parameters from 1464 reflections
*a* = 12.285 (2) Å	θ = 2.1–28.2º
*b* = 8.4576 (15) Å	µ = 0.09 mm^−^^1^
*c* = 15.6162 (19) Å	*T* = 293 (2) K
β = 127.566 (8)º	Block, yellow
*V* = 1286.1 (3) Å^3^	0.20 × 0.15 × 0.10 mm
*Z* = 4	

### Data collection

**Table:**

Bruker SMART CCD area-detector diffractometer	1464 reflections with *I* > 2σ(*I*)
Radiation source: fine-focus sealed tube	*R*_int_ = 0.060
Monochromator: graphite	θ_max_ = 28.2º
*T* = 293(2) K	θ_min_ = 2.1º
φ and ω scans	*h* = −16→13
Absorption correction: none	*k* = −10→11
8080 measured reflections	*l* = −19→20
3090 independent reflections	

### Refinement

**Table:**

Refinement on *F*^2^	Hydrogen site location: inferred from neighbouring sites
Least-squares matrix: full	H-atom parameters constrained
*R*[*F*^2^ > 2σ(*F*^2^)] = 0.056	*w* = 1/[σ^2^(*F*_o_^2^) + (0.0831*P*)^2^] where *P* = (*F*_o_^2^ + 2*F*_c_^2^)/3
*wR*(*F*^2^) = 0.178	(Δ/σ)_max_ < 0.001
*S* = 0.98	Δρ_max_ = 0.33 e Å^−^^3^
3090 reflections	Δρ_min_ = −0.21 e Å^−^^3^
173 parameters	Extinction correction: SHELXL97 (Sheldrick, 2008), Fc^*^=kFc[1+0.001xFc^2^λ^3^/sin(2θ)]^-1/4^
Primary atom site location: structure-invariant direct methods	Extinction coefficient: 0.051 (6)
Secondary atom site location: difference Fourier map	

### Special details

**Table:**

Geometry. All e.s.d.'s (except the e.s.d. in the dihedral angle between two l.s. planes) are estimated using the full covariance matrix. The cell e.s.d.'s are taken into account individually in the estimation of e.s.d.'s in distances, angles and torsion angles; correlations between e.s.d.'s in cell parameters are only used when they are defined by crystal symmetry. An approximate (isotropic) treatment of cell e.s.d.'s is used for estimating e.s.d.'s involving l.s. planes.
Refinement. Refinement of *F*^2^ against ALL reflections. The weighted *R*-factor *wR* and goodness of fit *S* are based on *F*^2^, conventional *R*-factors *R* are based on *F*, with *F* set to zero for negative *F*^2^. The threshold expression of *F*^2^ > σ(*F*^2^) is used only for calculating *R*-factors(gt) *etc*. and is not relevant to the choice of reflections for refinement. *R*-factors based on *F*^2^ are statistically about twice as large as those based on *F*, and *R*- factors based on ALL data will be even larger.

### Fractional atomic coordinates and isotropic or equivalent isotropic displacement parameters (Å^2^)

**Table:**

	*x*	*y*	*z*	*U*_iso_*/*U*_eq_	
O1	0.66997 (19)	−0.2663 (2)	−0.13413 (14)	0.0613 (6)	
O2	0.38437 (19)	−0.1191 (2)	−0.05358 (15)	0.0610 (6)	
N1	0.50763 (19)	−0.1704 (2)	−0.11721 (15)	0.0443 (5)	
N2	0.3078 (2)	−0.1271 (2)	−0.30016 (15)	0.0481 (6)	
N3	0.3112 (2)	−0.2458 (2)	−0.35815 (16)	0.0508 (6)	
C1	0.1467 (3)	0.0376 (4)	−0.2904 (2)	0.0729 (9)	
H1A	0.2309	0.0833	−0.2297	0.109*	
H1B	0.0965	−0.0058	−0.2672	0.109*	
H1C	0.0925	0.1178	−0.3439	0.109*	
C2	0.1782 (3)	−0.0900 (3)	−0.3378 (2)	0.0520 (7)	
C3	0.0937 (3)	−0.1884 (3)	−0.4239 (2)	0.0575 (7)	
H3B	−0.0016	−0.1922	−0.4669	0.069*	
C4	0.1783 (3)	−0.2813 (3)	−0.4344 (2)	0.0524 (7)	
C5	0.1404 (3)	−0.4075 (4)	−0.5153 (2)	0.0737 (9)	
H5A	0.2223	−0.4502	−0.5013	0.111*	
H5B	0.0833	−0.3632	−0.5867	0.111*	
H5C	0.0914	−0.4901	−0.5099	0.111*	
C6	0.4354 (2)	−0.0637 (3)	−0.20848 (18)	0.0486 (6)	
H6A	0.4176	0.0345	−0.1870	0.058*	
H6B	0.4937	−0.0397	−0.2292	0.058*	
C7	0.6197 (2)	−0.2631 (3)	−0.0875 (2)	0.0442 (6)	
C8	0.6612 (2)	−0.3498 (3)	0.01092 (18)	0.0460 (6)	
C9	0.7624 (3)	−0.4605 (3)	0.0711 (2)	0.0611 (8)	
H9A	0.8183	−0.4937	0.0531	0.073*	
C10	0.7773 (3)	−0.5210 (4)	0.1611 (2)	0.0708 (9)	
H10A	0.8446	−0.5966	0.2038	0.085*	
C11	0.6954 (3)	−0.4717 (4)	0.1882 (2)	0.0685 (9)	
H11A	0.7098	−0.5121	0.2498	0.082*	
C12	0.5915 (3)	−0.3625 (3)	0.12510 (19)	0.0561 (7)	
H12A	0.5342	−0.3306	0.1420	0.067*	
C13	0.5765 (2)	−0.3033 (3)	0.03664 (18)	0.0439 (6)	
C14	0.4762 (3)	−0.1880 (3)	−0.04554 (19)	0.0454 (6)	

### Atomic displacement parameters (Å^2^)

**Table:**

	*U*^11^	*U*^22^	*U*^33^	*U*^12^	*U*^13^	*U*^23^
O1	0.0654 (12)	0.0772 (13)	0.0586 (11)	0.0047 (10)	0.0467 (11)	−0.0022 (9)
O2	0.0650 (12)	0.0651 (12)	0.0718 (12)	0.0080 (10)	0.0515 (11)	0.0043 (10)
N1	0.0474 (12)	0.0503 (12)	0.0403 (11)	0.0032 (10)	0.0294 (10)	0.0019 (9)
N2	0.0492 (12)	0.0535 (13)	0.0422 (11)	0.0021 (10)	0.0282 (10)	0.0022 (10)
N3	0.0583 (14)	0.0535 (13)	0.0446 (12)	0.0000 (11)	0.0333 (12)	−0.0004 (10)
C1	0.0560 (18)	0.082 (2)	0.073 (2)	0.0076 (16)	0.0352 (16)	−0.0100 (17)
C2	0.0488 (15)	0.0574 (16)	0.0486 (14)	0.0033 (13)	0.0290 (13)	0.0018 (13)
C3	0.0465 (15)	0.0656 (18)	0.0539 (16)	0.0002 (14)	0.0272 (14)	−0.0010 (14)
C4	0.0558 (16)	0.0550 (16)	0.0448 (14)	−0.0043 (13)	0.0298 (14)	0.0034 (12)
C5	0.075 (2)	0.075 (2)	0.0697 (18)	−0.0117 (17)	0.0434 (17)	−0.0163 (17)
C6	0.0504 (15)	0.0524 (15)	0.0419 (14)	−0.0034 (12)	0.0276 (13)	−0.0003 (12)
C7	0.0455 (14)	0.0481 (14)	0.0425 (13)	−0.0036 (11)	0.0287 (12)	−0.0064 (11)
C8	0.0428 (14)	0.0493 (15)	0.0407 (13)	−0.0045 (12)	0.0227 (12)	−0.0046 (12)
C9	0.0532 (17)	0.0645 (18)	0.0576 (17)	0.0062 (14)	0.0297 (14)	0.0038 (15)
C10	0.0634 (19)	0.064 (2)	0.0615 (19)	0.0063 (15)	0.0261 (16)	0.0135 (15)
C11	0.078 (2)	0.072 (2)	0.0461 (16)	−0.0097 (17)	0.0329 (16)	0.0050 (15)
C12	0.0666 (18)	0.0576 (17)	0.0447 (14)	−0.0142 (14)	0.0343 (14)	−0.0067 (13)
C13	0.0479 (14)	0.0455 (14)	0.0375 (13)	−0.0073 (11)	0.0256 (12)	−0.0054 (11)
C14	0.0471 (14)	0.0521 (15)	0.0413 (13)	−0.0039 (12)	0.0292 (12)	−0.0051 (12)

### Geometric parameters (Å, °)

**Table:**

O1—O1	0.000 (5)	C5—H5A	0.9600
O1—C7	1.208 (3)	C5—H5B	0.9600
O2—C14	1.206 (3)	C5—H5C	0.9600
N1—C7	1.396 (3)	C6—H6A	0.9700
N1—C14	1.397 (3)	C6—H6B	0.9700
N1—C6	1.446 (3)	C7—O1	1.208 (3)
N2—C2	1.355 (3)	C7—C8	1.485 (3)
N2—N3	1.369 (3)	C8—C9	1.372 (3)
N2—C6	1.435 (3)	C8—C13	1.382 (3)
N3—C4	1.343 (3)	C9—C10	1.399 (4)
C1—C2	1.487 (4)	C9—H9A	0.9300
C1—H1A	0.9600	C10—C11	1.373 (4)
C1—H1B	0.9600	C10—H10A	0.9300
C1—H1C	0.9600	C11—C12	1.385 (4)
C2—C3	1.370 (4)	C11—H11A	0.9300
C3—C4	1.390 (4)	C12—C13	1.372 (3)
C3—H3B	0.9300	C12—H12A	0.9300
C4—C5	1.495 (4)	C13—C14	1.479 (4)
			
O1—O1—C7	0(10)	N1—C6—H6A	109.0
C7—N1—C14	111.9 (2)	N2—C6—H6B	109.0
C7—N1—C6	124.48 (19)	N1—C6—H6B	109.0
C14—N1—C6	123.6 (2)	H6A—C6—H6B	107.8
C2—N2—N3	112.6 (2)	O1—C7—O1	0.00 (8)
C2—N2—C6	128.8 (2)	O1—C7—N1	125.1 (2)
N3—N2—C6	118.6 (2)	O1—C7—N1	125.1 (2)
C4—N3—N2	103.8 (2)	O1—C7—C8	129.3 (2)
C2—C1—H1A	109.5	O1—C7—C8	129.3 (2)
C2—C1—H1B	109.5	N1—C7—C8	105.5 (2)
H1A—C1—H1B	109.5	C9—C8—C13	121.6 (2)
C2—C1—H1C	109.5	C9—C8—C7	129.9 (2)
H1A—C1—H1C	109.5	C13—C8—C7	108.5 (2)
H1B—C1—H1C	109.5	C8—C9—C10	116.5 (3)
N2—C2—C3	105.8 (2)	C8—C9—H9A	121.7
N2—C2—C1	123.1 (2)	C10—C9—H9A	121.7
C3—C2—C1	131.1 (2)	C11—C10—C9	121.8 (3)
C2—C3—C4	106.6 (2)	C11—C10—H10A	119.1
C2—C3—H3B	126.7	C9—C10—H10A	119.1
C4—C3—H3B	126.7	C10—C11—C12	120.9 (3)
N3—C4—C3	111.2 (2)	C10—C11—H11A	119.5
N3—C4—C5	119.5 (2)	C12—C11—H11A	119.5
C3—C4—C5	129.3 (2)	C13—C12—C11	117.4 (3)
C4—C5—H5A	109.5	C13—C12—H12A	121.3
C4—C5—H5B	109.5	C11—C12—H12A	121.3
H5A—C5—H5B	109.5	C12—C13—C8	121.7 (3)
C4—C5—H5C	109.5	C12—C13—C14	130.1 (2)
H5A—C5—H5C	109.5	C8—C13—C14	108.2 (2)
H5B—C5—H5C	109.5	O2—C14—N1	124.3 (2)
N2—C6—N1	112.99 (19)	O2—C14—C13	129.8 (2)
N2—C6—H6A	109.0	N1—C14—C13	105.9 (2)

### Hydrogen-bond geometry (Å, °)

**Table:**

*D*—H···*A*	*D*—H	H···*A*	*D*···*A*	*D*—H···*A*
C6—H6B···O1	0.97	2.58	2.917 (3)	101
C11—H11A···Cg2^i^	0.93	2.96	3.723 (3)	140

Symmetry codes:  (i) −*x*+1, −*y*−1, −*z*.
